# Roselle attenuates cardiac hypertrophy after myocardial infarction *in vivo* and *in vitro*

**DOI:** 10.17179/excli2019-1792

**Published:** 2019-09-26

**Authors:** Lislivia-Yiang-Nee Si, Anand Ramalingam, Shafreena Shaukat Ali, Amnani Aminuddin, Pei-Yuen Ng, Jalifah Latip, Yusof Kamisah, Siti Balkis Budin, Satirah Zainalabidin

**Affiliations:** 1 Programme of Biomedical Science, Centre of Applied and Health Sciences, Faculty of Health Sciences, Universiti Kebangsaan Malaysia, Jalan Raja Muda Abdul Aziz, 50300, Kuala Lumpur, Malaysia; 2 Drug and Herbal Research Center, Faculty of Pharmacy, Universiti Kebangsaan Malaysia, Jalan Raja Muda Abdul Aziz, 50300, Kuala Lumpur, Malaysia; 3 School of Chemical Sciences and Food Technology, Faculty of Science and Technology, Universiti Kebangsaan Malaysia, 43600, Bangi, Selangor, Malaysia; 4 Department of Pharmacology, Faculty of Medicine, Universiti Kebangsaan Malaysia, Jalan Yaacob Latif, 56000 Kuala Lumpur, Malaysia

**Keywords:** cardiac dysfunction, cardiomyocyte hypertrophy, fibrosis, myocardial infarction, oxidative stress, roselle

## Abstract

Roselle (*Hibiscus sabdariffa *Linn) has been traditionally used as folk medicine for hypertension and maintaining cardiovascular health, with therapeutic potential in protecting against numerous cardiovascular diseases. However, it remains unclear whether roselle can be used for management of cardiac hypertrophy seen after myocardial infarction (MI). This study therefore investigated the effects of aqueous roselle extract on cardiac hypertrophy arising from myocardial infarction both *in vivo* and *in vitro*. For *in vivo* study, male Sprague-Dawley rats were divided into control or MI groups (receiving 85 mg/kg isoproterenol s.c. for 2 days) and were given roselle extract (100 mg/kg, p.o daily) for 28 days. Cardiac structure and functional changes were evaluated at study end-point using histology, Langendorff analysis and gene expression analysis. *In vitro *effects of roselle were also assessed on ANG II-induced cardiomyocytes hypertrophy using H9c2 cells, simulating cardiac hypertrophy evident after MI. Roselle significantly ameliorated MI-induced cardiac systolic and diastolic dysfunction, as seen across improvement in left ventricular developed pressure (LVDP) and its derivative (LVdP/dt_max_) and isovolumic relaxation (Tau). Oxidative stress evident across elevated pro-oxidant markers (NOX2 subunit of NADPH oxidase and 8-isoprostane) as well as reduced antioxidant markers (superoxide dismutase and glutathione) were also significantly attenuated by roselle. Furthermore, roselle treatment markedly reduced markers of cardiac remodeling (cardiac hypertrophy and fibrosis) compared to the untreated MI rats. On *in vitro* analysis, roselle significantly attenuated ANG II-induced cardiomyoycte hypertrophy in dose-dependent manner. This study demonstrated that roselle attenuates cardiac hypertrophy and dysfunction seen after MI both *in vivo* and *in vitro*, and these effects are likely mediated by phenolic compounds found in roselle extract.

## Introduction

Cardiovascular disease is a major public health burden and its incidence is alarmingly increasing each year in developing and developed countries. Myocardial infarction (MI), characterized by necrosis of the heart muscle; prevails as the main cause of death from cardiovascular disease in Malaysia and worldwide. MI was constantly recorded as the primary cause of death in the past decade (from 2007-2017); with ~54 % increment in its incidence (Department of Statistics Malaysia, 2019[14]). Prolonged ischemia due to imbalances in oxygenated blood supply and demand causes acute necrosis in myocardium and results in MI. 

In order to compensate loss of functional cardiomyocytes, the heart therefore undergoes remodeling via a series of alterations in structure. Post-MI cardiac remodeling, specifically hypertrophy associated with fibrosis occurs at both molecular and celullar level and is mediated by oxidative stress (Prabhu and Frangogiannis, 2016[38]). The extent of cardiac remodeling is recognized as a strong predictor of mortality and morbidity among MI patients (Ali et al., 2019[3]; Azevedo et al., 2016[6]). Indeed, cardiac hypertrophy and fibrosis are early indications of progressive heart disease and increase the risk of heart failure, recurrent MI, arrhythmia and sudden death Azevedo et al., 2016[6]; Frey et al., 2004[19]).

Isoproterenol (ISO) is a synthetic catecholamine that exerts positive chronotropic and inotropic effects on the heart by binding to β-adrenoceptors and increasing calcium influx into cardiomyocytes. In large doses however, ISO causes greater myocardial oxygen demand, and insufficient oxygen supply to meet the demand that leads to eventual myocardial ischemia and MI (Zhang et al., 2016[55]). ISO-induced MI model is thus widely used by researchers nowadays to experimentally induce MI due to the reasons of having lower mortality rate and does not involve invasive procedure compared to other MI models such as coronary artery ligation (Lobo Filho et al., 2011[28]). Besides, the pathophysiological changes in the heart following ISO administration include increased cardiac injury markers enzyme activity, cardiac remodeling accompanied with systolic and diastolic dysfunctions, which closely resembles clinical conditions of MI in humans (Dianita et al., 2015[15]; Sidiqui et al., 2016[46]). *In vitro *model of ANG II induced cardiomyocyte hypertrophy has also been well documented (Kinobe et al., 2016[27]; Ni et al., 2018[31]; Watkins et al., 2012[50]). ANG II, as the primary effector peptide of the renin-angiotensin system plays a vital role in mediating cardiomyocyte hypertrophy via ANG II type 1 receptor, as commonly *in vivo* after MI (Ma et al. 2019[29]; Peng et al., 2016[37]). Collectively, these evidences justify the rationale of using *in vivo *ISO-induced MI model and *in vitro *ANG II induced cardiomyocyte hypertrophy model in present study.

Roselle or scientifically known as *Hibiscus sabdariffa *Linn., is a local tropical plant traditionally used as folk medicine for hypertension (Ali et al., 2005[2]). It is also commonly consumed as hot tea, juice, jam and jelly or used as food preservative. Several studies reported that roselle possesses a wide range of therapeutic effects, including antioxidant, antipyretic, antibacterial, anti-inflammatory, both antihyperglycemia and antihyperlipidemia in insulin-resistant rats, as well as reduced cardiac hypertrophy in hypertensive rats and patients (Ali et al., 2005[2]; Al-Shafei and El-Gendy, 2013[4]; Odigie et al., 2003[34]). Moreover, its role in hypertension is also well-documented, due to its rich source of high antioxidant polyphenols in its calyces. Recently, its cardioprotective effects on oxidative damage in diet-induced obese rats was established in our laboratory (Si et al., 2017[45]). In spite of all these evidences, little is known about the effects of roselle treatment on post-MI cardiac hypertrophy. Hence, this study was undertaken to look into the effects of roselle in both post-MI *in vivo* and *in vitro *cardiac hypertrophy. 

## Materials and Methods

### Roselle aqueous extract preparation

Dried roselle (*H. sabdariffa* Linn. UMKL-1) calyces were purchased on March 2015 from HERBagus Sdn Bhd in Kepala Batas, Penang, Malaysia with voucher specimen of PID050515-05, deposited at Forest Institute of Malaysia (FRIM). Roselle aqueous extract was prepared as previously described (Nnamonu et al., 2013[32]; Chumsri et al., 2008[12]). Dried calyces were ground with a blender (Cornell, Malaysia) and the powdered calyces were soaked in distilled water (1:10 ratio) for 24 h with occasional shaking. The mixture was then filtered with a piece of clean, sterile coarse cloth and the filtrate was kept frozen at -20 °C in an aluminium-wrapped plastic container overnight. The solidified roselle extract was freeze-dried (Labconco, US) and stored in a dark bottle at 4 °C until use.

### Identification and quantification of target compounds

For identification of phenolic compounds found in roselle extract, target compounds were analyzed by the high performance liquid chromatography (HPLC) method (Si et al., 2017[45]). Briefly, 10 mg of freeze-dried extract from roselle calyces was dissolved in 0.1 % (v/v) formic acid, sonicated for 5 min and filtered using a 0.45 μm polytetrafluoroethylene (PTFE) membrane syringe filter (Gema Medical, Spain). HPLC was then carried out using a HPLC Waters e2695 separation module equipped with a degasser (Waters, UK), an autosampler automatic injector and a Waters 2998 Photodiode Array Detector. The separation was performed using Purospher STAR RP-18e LichroCART column (250 mm x 4.6 mm x 5 µm particle size) (Merck Chemicals, Germany) at a flow rate of 1.0 mL/min, injection volume of 20 μL and 30 °C column oven temperature. 0.1 % formic acid in water and 0.1 % formic acid in acetonitrile were employed as mobile phases A and B respectively, in gradient elution as follows: 10-25 % B (0-5 min), 25-90 % B (15-20 min), 90-10 % B (25-25 min) and 10 % B (25-30 min). UV absorption was measured at 265 nm and 360 nm. Three compounds including ascorbic acid, chlorogenic acid and caffeic acid were used as standards. 

### Chemicals, drugs and reagents

Ascorbic acid, chlorogenic acid and caffeic acid standards were purchased from Extrasynthase, France. HPLC grade of solvents such as methanol and acetonitrile were obtained from Merck Chemicals, Germany while formic acid was obtained from Merck Chemicals, Finland. Isoproterenol hydrochloride, heparin and ANG II were obtained from Sigma Aldrich, USA while urethane was obtained from Merck Chemicals, USA. 3-(4,5-dimethylthiazol-zyl)-2,5-diphenyltetrazolium bromide (MTT) powder, dimethyl sulfoxide (DMSO), triton X-100, and formaldehyde were bought from Nacalai Tesque Inc, Japan. Cell staining reagents used included Alexa Fluor™ 488 phalloidin (Invitrogen, United Kingdom), 4',6-diamidino-2-phenylindole dihydrochloride (DAPI) (product no: 62248, Thermo Scientific, United States), and Dako fluorescent mounting medium (Agilent Technologies Inc, United States).

### Animal study

All procedures involving use of animals were subjected to approval from Universiti Kebangsaan Malaysia Animal Ethics Committee (FSK/2015/SATIRAH/11-FEB./644-FEB.-2015-SEPT.-2016). Male *Sprague-Dawley* rats (7-8 weeks; 250-300 g) were allowed to adapt to laboratory conditions for 1 week with *ad libitum* access to water and normal rat diet before the experiment started. Throughout the experiment, all rats were housed under same laboratory conditions of ambient room temperature and lighting (12 h light dark cycle).

The rats were randomly allotted into three groups with n=6 per group: control group (0.9 % normal saline); MI group (ISO, 85 mg/kg, subcutaneous, 2 days) (Ali et al., 2019[3]) and MI+R group which received roselle aqueous extract treatment (100 mg/kg, orally, 28 days) (Si et al., 2017[45]) via oral-force feeding after 24 h of second injection of ISO. MI was validated by increased plasma troponin-T (Elabscience Biotech Co Ltd, China) level (Supplementary Figure 1). Throughout the experiment, body weight gain was recorded at weekly intervals. Heart weight/tibia length (HW/TL) ratio was also recorded at the end of experiment as indices of cardiac hypertrophy.

### Blood pressure measurement 

Blood pressure (BP) was measured in conscious rats at the end of the experiment using non-invasive tail-cuff method (CODA 2-channel non-invasive blood pressure system, Kent Scientific Corporation, USA) as previously described (Si et al., 2017[45]). Each measurement session consisted of 5 acclimatization cycles followed by 15 BP measurement cycles. Systolic BP and heart rate (HR) measurements were recorded. 

### Langendorff analysis

For measurement of cardiac function, rat hearts were isolated and perfused *ex vivo* using Langendorff method (Yusof et al., 2018[53]). Rats were given heparin (500 U/kg, i.p.) to prevent blood clot in the heart and urethane (1 g/kg, i.p.) for anesthesia. After loss of pedal reflex activity, the heart was excised and placed in ice-cold Krebs-Henseleit buffer prior to cannulation of aorta to Langendorff apparatus. Each heart was perfused retrogradely in constant pressure mode (~80 mmHg) with Krebs-Henseleit buffer (in mM: NaCl 118.0; KCl 4.7; MgSO_4_ 1.2; NaHCO_3_ 25.0; KH_2_PO_4_ 1.2; CaCl_2_ 2.5; glucose 11.0) with pH 7.4 and was continuously aerated with 95 % O_2_ and 5 % CO_2_ at 37 °C. A water-filled latex balloon connected to pressure transducer (MLT844, ADInstrument, Australia) was inserted through mitral valve into the left ventricle to allow isovolumic contraction and measurement of intraventricular pressure changes. After 20 min of stabilization, cardiac function parameters such as left ventricular developed pressure (LVDP), left ventricular (LV) maximum and minimum rate of pressure changes (LVdP/dt_max _and LVdP/dt_min_) as well as the time constant of isovolumic relaxation (Tau) were assessed using a PowerLab data acquisition system. The rate of coronary flow was also recorded by measuring the amount of perfusate flow out from coronary in one minute, whereas rate of pressure product (RPP) was formulated from LVDP x HR. All data were acquired using chart software (LabChart 7.0, ADInstrument, Australia).

### Assessment of oxidative stress in heart homogenate 

A portion of heart tissue was collected at endpoint after Langendorff analysis and kept for biochemical studies and histology. Heart tissue was homogenized in lysis buffer (1:3 w/v ratio) and centrifuged at 12,000 rpm for 30 min at 4 °C for collection of heart homogenate. 8-isoprostane level in homogenate was measured using a commercial ELISA kit (Elabscience Biotech Co Ltd, China) according to manufacturer guidelines. Endogenous antioxidant status in heart homogenate was also determined by measuring superoxide dismutase (SOD) enzyme activity (Beyer and Fridovich, 1987[7]) and reduced glutathione (GSH) concentration (Ellman, 1959[16]) using colorimetric assay methods. 

### Histological analysis

Heart tissue was fixed in 10 % formalin and embedded into paraffin blocks. Paraffin-embedded tissues were sectioned into 4 µm thickness and stained with Hematoxylin and Eosin (H&E) to measure size of cardiomyocytes or picrosirius red to estimate percentage of collagen deposition as previously described (Ali et al., 2019[3]). Quantitative measurements of cardiomyocyte cross-sectional area (circumferential length of myocytes) and percentage of collagen deposition was calculated using ImageJ software (Bethesda, Maryland, USA) (Ali et al., 2019[3]).

### Gene expression analysis

Total RNA was extracted from frozen heart tissues and reverse transcribed using previously described methodology (Ali et al., 2019[3]). Gene expression of oxidative stress marker (NOX2 subunit of NADPH oxidase, NOX2), hypertrophy markers (atrial natriuretic peptide, ANP and brain natriuretic peptide, BNP), and fibrosis markers (collagen 1, Col 1 and collagen 3, Col 3) were measured via quantitative real time-PCR using SYBR® Green (Applied Biosystems, Scoresby, Victoria, Australia). Ribosomal 18S gene expression was used as the endogenous control. Primers were generated from rat sequences in GenBank. Primer sequences are given in online-only supplement (Supplementary Table 1). Quantitative analysis was performed using ABI Prism® 7700 Sequence Detection software, using the ΔΔCt method to detect fold differences relative to the control group.

### Cell culture

H9c2 cardiomyocyte cell line was purchased from American Type Culture Collection (ATCC) and cultured in Dulbecco's Modified Eagle's Medium with high glucose (DMEM, high glucose) (Life Technologies Corporation, USA) containing 10 % fetal calf serum (FCS) (GE Healthcare Life Sciences, USA) and 1 % penicillin/streptomycin (Nacalai Tesque Inc., Japan). The cells were maintained in a humidified incubator with 5 % CO_2_ at 37 °C. All experiments were conducted in three independent replicates.

### Cytotoxicity effects of roselle extract

Cytotoxicity effect of roselle extract on H9c2 cardiomyocytes was evaluated using MTT assay. Cells were seeded at a density of 5 x 10^3^ cell/well in a 96-well plate (Nest Biotech Co., Ltd, China) and left overnight for cell attachment prior to treatment with roselle extract at varying concentrations (0.3125 - 15.0 mg/mL). After 24 h, MTT solution (0.5 mg/mL) was added and the cells were further incubated for 4 h before addition of DMSO for solubilization of formazan crystals produced by viable cells. The absorbance was measured at a wavelength of 570 nm with Infinite® 200 PRO microplate reader (Tecan Group Ltd., Switzerland). 

### In vitro effects of roselle extract on hypertrophic cardiomyocytes

Cells were seeded at density 1 x 10^4^ cells/ml on coverslips in 6-well plates (Nest Biotech Co., Ltd, China) and left overnight for cell attachment in 5 % CO_2_ incubator at 37 °C. Final concentration of 200 nM ANG II (Hernandez et al., 2014[22]) (determined using preliminary analysis, Supplementary Figure 2) was used to induce hypertrophy. Briefly, cells were treated with roselle extract 6 h before, simultaneously with, and 6 h after the addition of ANG II for pre-, simultaneous, and post-treatment groups respectively. Each experimental design group consisted of control, 10 µg/mL roselle-treated only, ANG II-treated only, and both ANG II- and roselle-treated cells with varying concentrations (1, 5, 10 µg/mL) groups. All groups were further cultured for an additional 24 h prior to immunofluorescence staining. The cells were washed with phosphate-buffered saline (PBS), permeabilized with 0.1 % v/v Triton X-100 in PBS and fixed with 3.7 % v/v formaldehyde in PBS. The cells were subsequently stained with Alexa Fluor™ 488 phalloidin (1:40) and DAPI (1:1000). Images of the stained cells were captured using EVOS FL Autoimaging system at 200x magnification. The area of 10 individual cells from 10 fields was measured randomly per experimental group using ImageJ version 1.51k (National Institute of Health, USA). 

### Statistical analysis

Values from each group are presented as mean ± standard error (SEM). Statistical significance was determined using one-way analysis of variance (ANOVA) with Tukey's post-hoc test to analyze differences between groups with GraphPad Prism software version 6. A p-value of less than 0.05 was considered as statistically significant. 

## Results

### Target compounds characterised from roselle aqueous extract

**Figure 1:**
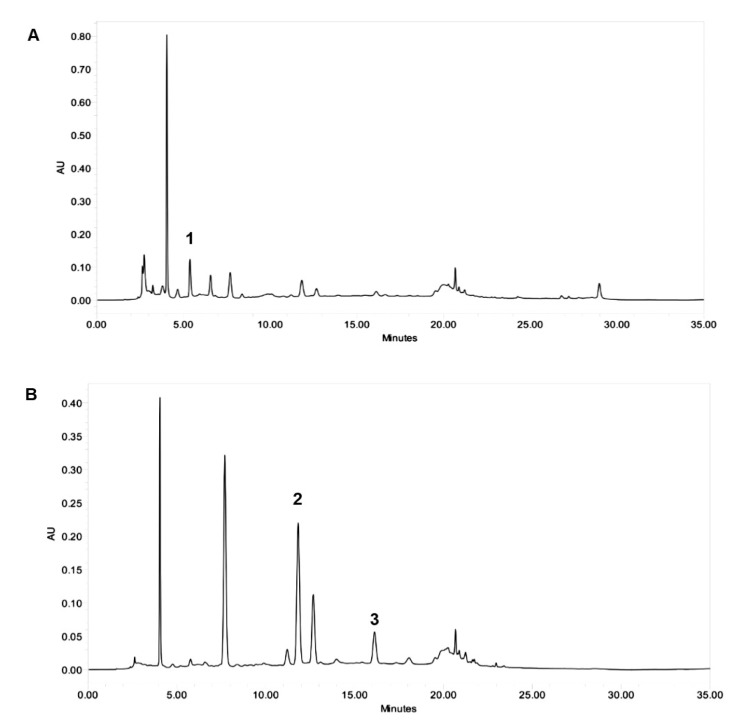
HPLC chromatograms of roselle aqueous extract recorded at (A) 265 nm and (B) 320 nm. Quantification showed that roselle aqueous extract contained (1) 13.12 ± 1.07 mg/g FED ascorbic acid (5.39 min), (2) 4.06 ± 0.3 mg/g FDE chlorogenic acid (11.91 min), and (3) 1.58 ± 0.01 mg/g FDE caffeic acid (16.29 min).

Three target compounds: ascorbic acid, chlorogenic acid and caffeic acid were detected from roselle extract by HPLC. Identification of each single compound was mainly based on HPLC retention time (RT) and matching UV absorption spectrum with standard compounds at 295 nm (for ascorbic acid) and 320 nm (for chlorogenic acid and caffeic acid). Target compounds quantification were determined by standard reference calibration curves and were expressed as mg per g freeze dried extract (FDE). Linear correlation co-efficient was > 0.996 for each compound. The result showed that roselle calyces contained ascorbic acid (RT: 5.39 min), chlorogenic acid (RT: 11.91 min) and caffeic acid (RT: 16.29 min) with 13.12 ± 1.07 mg/g FDE, 4.06 ± 0.3 mg/g FDE and 1.58 ± 0.01 mg/g FDE respectively (Figure 1(Fig. 1)).

### Effects of roselle on systemic characteristics in rats

MI significantly increased HW/TL ratio, systolic BP and HR in rats compared to the control group (Table 1(Tab. 1)). Roselle treatment, however, significantly attenuated all these systemic characteristics except for the HW/TL value. Body weight gain was unaltered by both MI and roselle treatment (Table 1(Tab. 1)). 

### Roselle supplementation ameliorates cardiac dysfunction post-MI in rats

**Figure 2:**
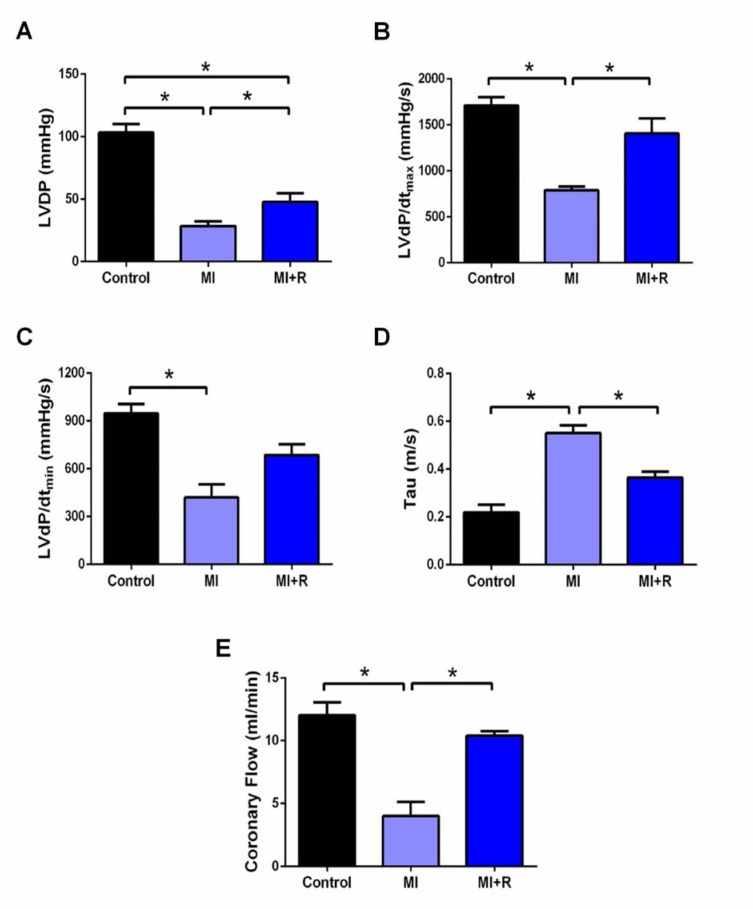
Roselle supplementation ameliorates cardiac dysfunction post-MI. (A) LVDP, (B) LVdP/dtmax, (C) LVdP/dtmin, (D) Tau, and (E) coronary flow. Data are presented as mean ± SEM for n=6 per group. ^*^p < 0.05 using one-way ANOVA with Tukey post-hoc test

MI group showed a significant deterioration in cardiac systolic function (LVDP and LVdP/dt_max_), diastolic function (LVdP/dt_min_ and Tau) and coronary flow rate compared to the control group (Figure 2A-E(Fig. 2)). However, roselle treatment effectively ameliorated cardiac systolic dysfunction and rescued coronary flow rate in these MI+R rats (Figure 2A-B and E(Fig. 2)). Treatment with roselle also significantly ameliorates cardiac diastolic dysfunction as shown across improved Tau (Figure 2C(Fig. 2)) besides also showed a statistically non significant tendency restoration of LVdP/dt_min_, p=0.06 (Figure 2D(Fig. 2)).

### Roselle supplementation attenuates cardiac oxidative stress post-MI in rats

**Figure 3:**
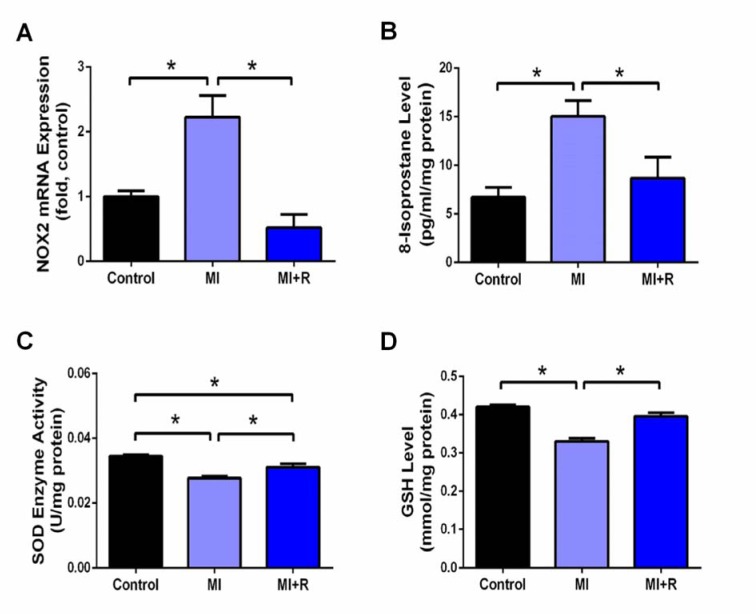
Roselle supplementation attenuates cardiac oxidative stress post-MI. (A) Nox2 fold changes in gene expression, relative to housekeeper 18S, measurement of oxidative stress marker, (B) 8-isoprostane and level of endogenous antioxidants (C) SOD and (D) GSH. Data are presented as mean ± SEM for n=6 per group. ^*^p < 0.05^*^p < 0.05 using one-way ANOVA with Tukey post-hoc test

MI induction significantly increased oxidative stress, as shown across significantly upregulated NOX2 gene expression and 8-isoprostane level, together with decreased endogenous antioxidants SOD activity and GSH concentration in the MI rat hearts compared to the controls. Roselle supplementation significantly attenuated all these changes (Figure 3A-D(Fig. 3)). 

### Roselle supplementation limits cardiac remodeling post-MI in rats

**Figure 4:**
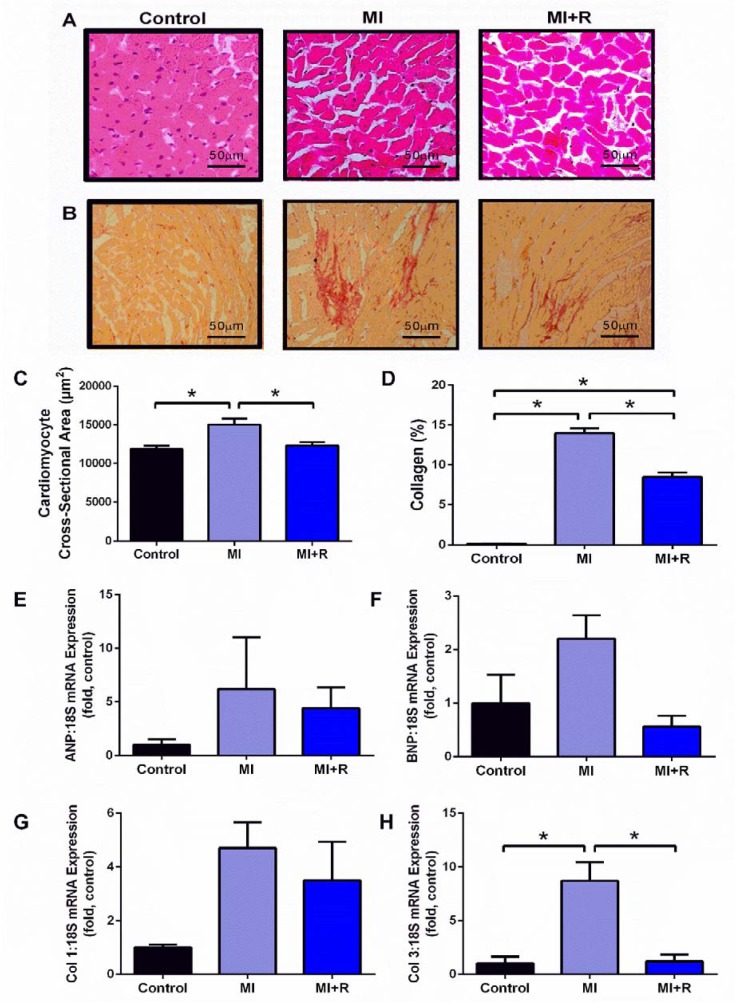
Roselle supplementation limits cardiac remodeling post-MI. Representative images from microscopic observation of heart tissue for each group with (A) H&E staining; 40x magnification, (B) picrosirius red staining; 10x magnification. Measurement were taken from each group for (C) cardiomyocytes cross-sectional area from H&E staining, (D) percentage of collagen deposition from picrosirius red staining. Fold changes in gene expression of (E) ANP, (F) BNP, (G) COL1, (H) COL3 relative to the housekeeper 18S. Data are presented as mean ± SEM for n=6 per group. ^*^p < 0.05 using one-way ANOVA with Tukey post-hoc test

Representative images of H&E stained & picrosirius red stained heart sections are shown in Figure 4A and 4B(Fig. 4) respectively. MI-subjected rats showed increased cardiomyocyte size (Figure 4C(Fig. 4)) and markedly increased interstitial collagen deposition compared to control rat hearts (Figure 4D(Fig. 4)). Gene expression of myocyte hypertrophy markers ANP (Figure 4E(Fig. 4)) and BNP (Figure 4F(Fig. 4)) were not significantly affected by both MI and roselle treatment. However, a trend for upregulation in gene expression of Col 1 and Col 3, suggestive of cardiac fibrosis was seen in MI group. Interestingly, treatment with roselle for 28 days markedly reduced the increment of cardiomyocytes cross-sectional area (Figure 4C(Fig. 4)), collagen deposition (Figure 4D(Fig. 4)) and gene expression of Col 3 (Figure 4H(Fig. 4)). 

### Roselle extract attenuates ANG II-induced cardiomyocyte hypertrophy in vitro

**Figure 5:**
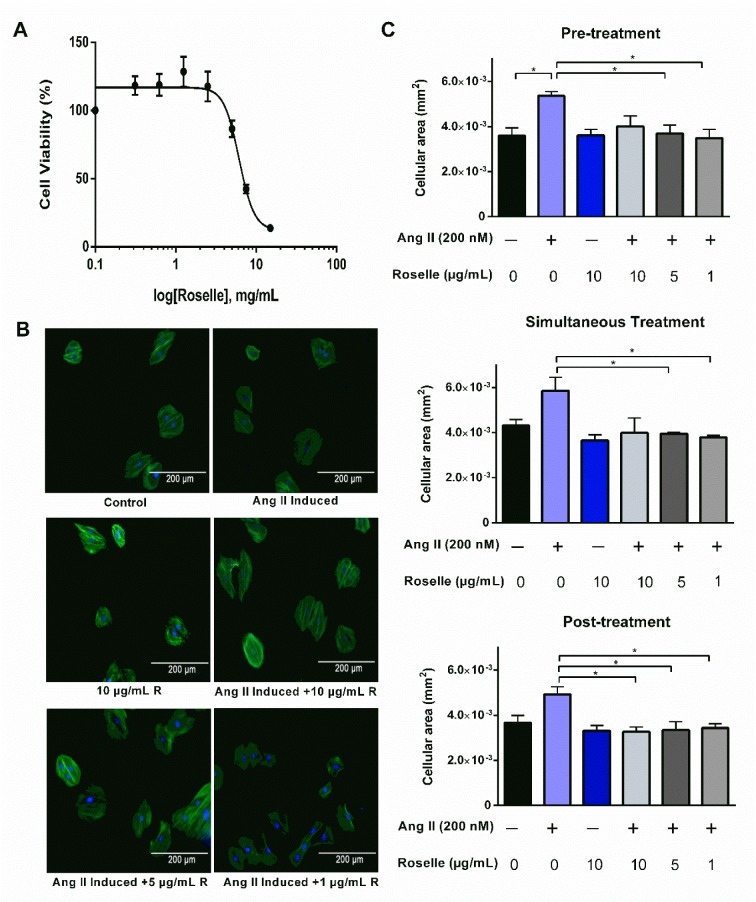
Roselle extract attenuates ANG II-induced cardiomyocyte hypertrophy. (A) Percentage of cell viability of H9c2 cell line against roselle extract at concentration of 0.3125, 0.6125, 1.25, 2.5, 5, 7.5, 10, and 15 mg/mL; concentrations of roselle are presented in log concentration, (B) Representative images from immunofluorescence microscopy of each experimental group where blue (DAPI) and green (Alexa Fluor™ 488 phalloidin) stains represent nucleus and cytoskeleton actin of the cells respectively; 200x magnification, (C) Average cellular area of each experimental group in pre-, simultaneous, and post-treatment experiments. Data are presented as mean ± SEM for n=3, 10 measurements of cell per group. *p < 0.05 using one-way ANOVA

Although treatment with roselle extract reduced cell viability of H9c2 cells in a dose-dependent manner, roselle extract at low doses showed no cytotoxic effects (Figure 5A(Fig. 5)). Based on the analysis, roselle extract at 1, 5 and 10 µg/mL were chosen for *in vitro* hypertrophy assay. H9c2 cells were treated with roselle for three different treatment conditions i.e. before, simultaneously, and after administration of ANG II and were observed through immunofluorescence microscopy after 24 h (Figure 5B(Fig. 5)). From the findings, ANG II increased cellular area of H9c2 cells in comparison to the control group, indicating hypertrophic effects. Both pre- and simultaneous treatment with roselle at 5 and 10 µg/ml caused a significant reduction in the average cellular area. Meanwhile, post-treatment of roselle at all three concentrations showed a significant reduction in average cellular area compared to the ANG II-treated group (Figure 5C(Fig. 5)).

## Discussion

We herein present our novel findings on the effects of roselle aqueous extract on post-MI cardiac hypertrophy in rats and on ANG II-induced hypertrophic cardiomyocytes. Cardiac remodeling is the fundamental process that characterizes progression of heart failure in MI patients. This complex process is closely linked with oxidative stress that precedes cardiac hypertrophy and fibrosis. Myocardial oxidative stress triggers cardiac structural alterations and contributes to the deterioration of cardiac function. Thus, any means that aim on limiting the structural remodeling may be an appropriate strategy to improve prognosis of patients with heart failure and prevent MI complications (Goldstein, 2006[20]).

We have shown in our laboratory that in animal models, cardiac remodeling after MI may occur as early as 7 days (Ali et al., 2019[3]) and is shown to decompensate into cardiac dysfunction within 4 weeks after injury (Si et al., 2017[45]). In the present study, *in vivo* ISO-induced MI model demonstrated that cardiac dysfunction was accompanied by features of excessive oxidative stress, cardiac hypertrophy associated with fibrosis. Roselle supplementation for 28 days successfully modulated cardiac remodeling by restoration of cardiac function, amelioration of cardiac oxidative stress, limiting cardiomyocytes hypertrophy and cardiac fibrosis. We have previously reported that acute administration of ISO produced similar myocardial aberrations with features of inflammation, hypertrophy accompanied by fibrosis and roselle supplementation for 7 days was able to modulate all these changes (Ali et al., 2019[3]). It is suggested that these effects are presumably contributed by presence of various bioactive compounds in roselle. Previous phytochemical reports have shown that roselle possesses bioactive compounds such as anthocyanins (Si et al., 2017[45]; Wu et al., 2018[52]), ascorbic acid, caffeic acid and chlorogenic acids (Prenesti et al., 2007[39]; Rababah et al., 2011[40]; Rodriguez-Medina et al., 2009[43]). Similarly, we also have successfully identified these compounds in our aqueous roselle extract. Several lines of evidence have shown cardioprotective capacity of these reported compounds in various kinds of animal models, hence justifying the positive effect of roselle seen in this present study (Bhandarkar et al., 2019[9]; Castellano et al., 2016[10]; Hao et al., 2016[21]). 

Consistent with previous studies (Riba et al., 2017[42]; Van Sligtenhorst et al., 2012[49]), the current study showed that rats following MI exhibited increment in HW/TL ratio where this increment could be contributed by increase in water content and cellular infiltration with elevation of protein in the damaged myocardium (Patel et al., 2010[36]; Upaganlawar et al., 2011[48]). Roselle supplementation for 28 days showed a trend of lowering the ratio of HW/TL, however, this reduction was not statistically significant, similar to our previous report (Si et al., 2017[45]). Besides, accumulating evidence has shown that ISO-induced MI is associated with high BP and HR (Bhandari et al., 2008[8]; Khorrami et al., 2014[26]; Nwokocha et al., 2017[33]). ISO exerts positive inotropic and chronotropic, contributing to raised BP and HR. Similarly, we also found that ISO administration elevated systolic BP and HR. Interestingly, roselle was able to normalize these hemodynamic changes seen in MI rats. This was consistent with our previous findings where chronic roselle supplementation modulated systolic BP in nicotine-induced cardiac injured rats (Ramalingam et al., 2016[41]), suggesting roselle possesses anti-hypertensive property. Past study suggested that roselle was able to lower BP via both endothelium-dependent and independent vasodilator pathways (Ajay et al., 2007[1]). Endothelium-dependent vasodilator involves activation of endothelium-derived nitric oxide/cGMP-relaxant pathway while endothelium independent pathway involves inhibition of Ca^2+^ entry into myocardium. 

In clinical settings, extensive post-MI cardiac remodeling promotes cardiac dysfunction which predisposes patients to developing heart failure. Apart from the systemic changes, rats subjected to MI also demonstrated depressed cardiac function as indicated by a fall in systolic and diastolic function, together with reduction in coronary flow. Improvement of systolic function by roselle was evident with the rise seen across LVDP and its derivatives LVdP/dt_max_, suggesting that roselle was able to limit left ventricular contractile dysfunction. Furthermore, roselle reduced stiffening of left ventricle chamber, as shown by positive alteration in LVdP/dt_min _and Tau value. Roselle also rescued coronary flow rate in MI rats, proving roselle is beneficial in improving cardiac function. Previous study has reported that administration of caffeic acid, a constituent which is also present in roselle effectively ameliorated cardiac dysfunction in MI rats (Kanno et al., 2013[25]) which could explain the beneficial effect of roselle on cardiac function seen in this study.

A plethora of evidence has reported that oxidative stress mediates damage seen in ISO-induced MI, which then initiates cardiac remodeling (Khorrami et al., 2014[26]; Panda et al., 2017[35]). Oxidative stress is characterized by a marked elevation in free radical production and suppression of antioxidant status. Marked upregulation of 8-isoprostane, a product of lipid peroxidation and NOX2 gene expression was seen following MI. Consistently, marked reduction in antioxidants such as SOD and GSH was also observed, suggestive of oxidative stress. Being a major source of reactive oxygen species (ROS), NOX2 upregulation initiates peroxidation of lipid, hence contributes to the buildup of 8-isoprostane (Si et al., 2017[45]). Indeed, cardiac tissue itself is a rich source of ROS, and NOX2 is a critical determinant of myocardial ROS generation (Frantz et al., 2006[18]). Interestingly, roselle being a powerful antioxidant as widely reported (Si et al., 2017[45]; Yusof et al., 2018[53]; Ramalingam et al., 2016[41]; Mohamed et al., 2013[30]; Zainalabidin et al., 2016[54]) was able to attenuate all these changes. It could be attributable to bioactive compounds characterized in our roselle aqueous extract where antioxidant capacity of ascorbic acid, caffeic acid and chlorogenic acid has been proven in various animal models. On top of that, chlorogenic acid was previously shown to scavenge free radicals, exhibiting SOD-like activity (Fernández-Arroyo et al., 2012[17]). 

Evidence from literature also demonstrated that alteration of cardiac structure due to fibrosis and hypertrophy plays a fundamental role in pathophysiology of heart failure in post-MI setting (Amoni et al., 2017[5]; Hori and Nishida, 2008[23]; Prabhu and Frangogiannis, 2016[38]; Talman and Ruskoaho, 2016[47]). Changes in ventricular mass, composition and volume may result in adverse outcome for cardiac function. Extensive deposition of collagen and extracellular matrix end up stiffening and altering physiological function of the heart. Col 1 and Col 3 are subtypes of collagen that are majorly found in cardiac fibrosis (Collier et al., 2012[13]). Upregulation of Col 1 and Col 3 gene expression was observed in MI subjected rats, similarly to our previous study (Ali et al., 2019[3]). Cardiac hypertrophy which initially starts as compensatory process may contribute to cardiac function deterioration by dilation of heart chamber. Consistent with past studies (Ali et al., 2019[3]; Chen et al., 2018[11]; Sagor et al., 2015[44]), we have also shown that rats subjected to MI exhibited features of cardiac hypertrophy and fibrosis, explaining the cardiac systolic and diastolic dysfunction reported earlier. Intense scarring of myocardium was evident through histopathological observation. On the other hand, increased size of cardiomyocytes suggested cardiac hypertrophy. Roselle treatment for 28 days successfully ablated deposition of myocardium collagen besides its ability to reduce the enlargement of cardiomyocytes. These findings are similar to our previous studies which showed that roselle supplementation was able to limit cardiac remodeling by modulating fibrosis and hypertrophy (Ali et al., 2019[3]; Si et al., 2017[45]). To further support our finding, it is also noteworthy to mention that ascorbic, caffeic acid (Weiskirchen, 2016[51]) and chlorogenic acid (Kanno et al., 2013[25]) have been previously reported to have anti-fibrotic activity in rat model of MI and hepatic cirrhosis.

Cardiac hypertrophy is considered as an important predictor for heart failure and mortality in patients surviving from MI. Therefore, *in vitro* cardiac hypertrophy model was developed by using ANG II, a potent vasoconstrictor, on H9c2 cell line to examine the *in vitro* cardioprotective effects of roselle extract. Prior to investigating the antihypertrophic effects of roselle, the cytotoxic effect of roselle extract on H9c2 cells was evaluated by measuring cell viability after 24 h exposure to certain range of concentrations. The findings showed that roselle affected cell viability of H9c2 cells where roselle concentration up to 5 mg/mL was safe towards H9c2 cells. The roselle concentrations that were being used in subsequent evaluation of roselle's antihypertrophic effects are slightly lower in order to investigate its potency.

Herein, this study reported the cardioprotective potential of roselle at a relatively low dose towards ANG II-induced cardiac hypertrophy in H9c2 cells, thus implying the high potency of roselle as antihypertrophic agent. The findings suggested that roselle treatment was able to prevent and reverse cardiac hypertrophy as it reduced the average cellular area when given before, upon as well as after induction of cardiomyocyte hypertrophy with ANG II. In agreement with several reports, roselle treatment has been reported to reverse hypertrophy in hypertensive rats and human (Al-Shafei and El-Gendy, 2013[4]; Odigie et al., 2003[34]; Nnamonu et al., 2013[32]; Inuwa et al., 2012[24]). Though cellular area was reduced significantly in pre-treatment as well as in simultaneous treatment, the concentrations needed to limit myocyte enlargement were only 5 and 10 µg/ml. Whereas the post-treatment of roselle group showed significant reduction of cellular area for all three concentrations (1, 5, 10 µg/ml). These results were in parallel with the *in vivo* results. Altogether, these results support roselle of having the capability to reverse hypertrophy effect, may it be *in vivo* or *in vitro*.

## Conclusion

Cardiac dysfunction after MI, as evidenced by deterioration of systolic and diastolic cardiac function were further accompanied by structural changes such as hypertrophy, fibrosis and oxidative stress. Roselle supplementation markedly restored these deleterious post-MI changes in cardiac function by limiting cardiac oxidative stress, hypertrophy and fibrosis. Moreover, this study also highlighted the ability of the roselle to attenuate *in vitro* ANG II-induced hypertrophy model, indicating the cardioprotective potential of roselle against cardiac hypertrophy. Given the diversity of bioactive compounds found in roselle aqueous extract which could contribute to various mechanisms, this warrants further investigation in searching of exact responsible mechanisms involved in cardioprotective effects of roselle. 

## Acknowledgement

This project was funded by NKEA Research Grant Scheme (NRGS) from Ministry of Agriculture, Malaysia (NH1014D061), Malaysia and Geran Universiti Penyelidikan from Universiti Kebangsaan Malaysia, (GUP-2017-018). We are thankful to the Faculty of Pharmacy, UKM for lending their Langendorff apparatus, Miss Rumaisak Farizal and Miss Nur Ain Fitria Mohd Rezazali for their assistance in animal handling and harvesting the cells and Miss Siti Aishah Mohd Ali for her technical assistance in HPLC.

## Conflict of interest

The authors declare that there is no conflict of interest regarding the publication of this paper.

## Authors' contributions

L.S.Y.N performed all the experiments, analyzed and interpreted the data and was involved in writing the manuscript. A.R was involved in designing and conducting the PCR experiments. S.S.A was involved in writing the mansucript. A.A, N.P.Y and Y.K. were involved in *in vitro* part of the study. J.L was involved in the phytochemical analysis. S.B.B co-supervised the work while S.Z supervised the whole work including designing the experiments and data analysis. All authors participated in the preparation of the manuscript and approved the final form of the manuscript. 

## Supplementary Material

Supplementary material

## Figures and Tables

**Table 1:**
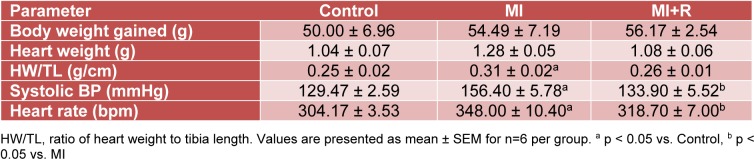
Systemic characteristics of the rats
