# Supplementary material for: Roselle attenuates cardiac hypertrophy after myocardial infarction in vivo and in vitro
Source: EXCLI J. 2019 Sep 26;18:876–92. doi: 10.17179/excli2019-1792 (PMC6806200; doi:10.17179/excli2019-1792)
Supplement: Supplementary material [file EXCLI-18-876-s-001.pdf]

EXCLI Journal 2019;18:876-892 – ISSN 1611-2156
Received: September 06, 2019, accepted: September 23, 2019, published: September 26, 2019

Supplementary material to:

ROSELLE ATTENUATES CARDIAC HYPERTROPHY AFTER MYOCARDIAL INFARCTION *IN VIVO* AND *IN VITRO*

Lislivia-Yiang-Nee Si[a], Anand Ramalingam[a], Shafreena Shaukat Ali[a], Amnani Aminuddin[c], Pei-Yuen Ng[c], Jalifah Latip[b], Yusof Kamisah[d], Siti Balkis Budin[a], Satirah Zainalabidin[a *]

[a] Programme of Biomedical Science, Centre of Applied and Health Sciences, Faculty of Health Sciences, Universiti Kebangsaan Malaysia, Jalan Raja Muda Abdul Aziz, 50300, Kuala Lumpur, Malaysia
[b] School of Chemical Sciences and Food Technology, Faculty of Science and Technology, Universiti Kebangsaan Malaysia, 43600, Bangi, Selangor, Malaysia
[c] Drug and Herbal Research Center, Faculty of Pharmacy, Universiti Kebangsaan Malaysia, Jalan Raja Muda Abdul Aziz, 50300, Kuala Lumpur, Malaysia
[d] Department of Pharmacology, Faculty of Medicine, Universiti Kebangsaan Malaysia, Jalan Yaacob Latif, 56000 Kuala Lumpur, Malaysia

* **Corresponding author:** Programme of Biomedical Science, Centre of Applied and Health Sciences, Faculty of Health Sciences, Universiti Kebangsaan Malaysia, Jalan Raja Muda Abdul Aziz, 50300, Kuala Lumpur, Malaysia. Tel No: +603-9289 7684, Fax No: +603-2692 9032, E-mail: satirah@ukm.edu.my

http://dx.doi.org/10.17179/excli2019-1792

This is an Open Access article distributed under the terms of the Creative Commons Attribution License (http://creativecommons.org/licenses/by/4.0/).

Supplementary Table 1: Primer sequences used in gene expression study

Gene	Forward (5'-3')	Reverse (5'-3')
18S	TTCGAGGCCCTGTAATTGGA	GCAGCAACTTTAATATAGGCTATTGG
ANP	GGAAGTCAACCCGTCTCAGA	TGGGCTCCAATCCTGTCAAT
BNP	ACAAGAGAGAGCAGGACACC	TCTGGAGACTGGCTAGGACT
Col 1	TGCTGCCTTTTCTGTTCCTT	AAGGTGCTGGGTAGGGAAGT
Col 3	GTCCACGAGGTGACAAAGGT	CATCTTTTCCAGGAGGTCCA

EXCLI Journal 2019;18:876-892 – ISSN 1611-2156
Received: September 06, 2019, accepted: September 23, 2019, published: September 26, 2019

Supplementary Table 2: Raw data to body weight gained, heart weight, heart weight/tibia length, systolic blood presurre (SBP) and heart rate (HR) of the rats (Table 1, main document); n=5-6; outliers were excluded based on GraphPad QuickCalcs.

Body weight gained (g)			
	**Control**	**MI**	**MI+R**
**n1**	68.00	27.8	58.0
**n2**	65.0	64.4	50.0
**n3**	41.0	74.0	56.0
**n4**	22.0	66.4	50.0
**n5**	48.0	39.8	58.0
**n6**	56.0	54.5	49.0
**Heart weight (g)**			
	**Control**	**MI**	**MI+R**
**n1**	1.01	1.14	1.24
**n2**	1.39	1.44	0.81
**n3**	0.94	1.13	1.11
**n4**	0.88	1.33	1.13
**n5**	0.98	1.38	1.12
**n6**	1.02	1.25	1.09
**Heart weight/Tibia length (g/cm)**			
	**Control**	**MI**	**MI+R**
**n1**	0.224	0.265	0.282
**n2**	0.331	0.360	0.189
**n3**	0.219	0.263	0.264
**n4**	0.215	0.309	0.282
**n5**	0.239	0.337	0.273
**n6**	0.243	0.298	0.273
**Systolic blood pressure (mmHg)**			
	**Control**	**MI**	**MI+R**
**n1**	131.0	136.0	144.5
**n2**	132.0	144.7	139.5
**n3**	117.0	153.7	131.4
**n4**	132.7	166.0	116.2
**n5**	129.5	173.0	150.8
**n6**	134.7	165.0	121.0
**Heart rate (bpm)**			
	**Control**	**MI**	**MI+R**
**n1**	315	313	328
**n2**	307	341	325
**n3**	311	371	300
**n4**	301	348	295
**n5**	291	367	326
**n6**	300		338

EXCLI Journal 2019;18:876-892 – ISSN 1611-2156
Received: September 06, 2019, accepted: September 23, 2019, published: September 26, 2019

Supplementary Table 3: Raw data to left ventricular developed pressure (LVDP), left ventricular (LV) maximum and minimum rate of pressure changes (LVdP/dtmax and LVdP/dtmin), time constant of isovolumic relaxation (Tau) and coronary flow. (Figure 2, main document); n=4-6; outliers were excluded based on GraphPad QuickCalcs.

LVDP (mmHg)			
	**Control**	**MI**	**MI+R**
**n1**	92.69	27.80	55.95
**n2**	81.53	26.17	62.80
**n3**	113.06	29.13	33.34
**n4**	121.18	45.82	38.90
**n5**	118.0	21.02	
**n6**	93.04	20.98	
**$LVdP/dt_{max}$ (mmHg/s)**			
	**Control**	**MI**	**MI+R**
**n1**	1770.08	859.89	1290.89
**n2**	1293.35	772.77	1175.01
**n3**	1854.45	752.84	1896.74
**n4**	1848.96	891.53	1259.31
**n5**	1685.26	634.73	
**n6**	1815.28	828.82	
**$LVdP/dt_{min}$ (mmHg/s)**			
	**Control**	**MI**	**MI+R**
**n1**	919.75	771.42	754.24
**n2**	766.36	362.55	503.43
**n3**	807.26	359.71	672.66
**n4**	1059.20	534.85	815.36
**n5**	1111.32	242.37	
**n6**	1027.07	243.48	
**Tau (m/s)**			
	**Control**	**MI**	**MI+R**
**n1**	0.31	0.46	0.39
**n2**	0.16	0.67	0.31
**n3**	0.14	0.57	0.33
**n4**	0.27	0.56	0.42
**n5**	0.15	0.58	
**n6**	0.29	0.46	
**Coronary flow (ml/min)**			
	**Control**	**MI**	**MI+R**
**n1**	15.51	0.80	9.39
**n2**	7.90	3.00	11.00
**n3**	13.03	1.91	10.48
**n4**	13.01	3.61	11.72
**n5**	11.15	7.93	
**n6**	11.49	6.72	

EXCLI Journal 2019;18:876-892 – ISSN 1611-2156
Received: September 06, 2019, accepted: September 23, 2019, published: September 26, 2019

Supplementary Table 4: Raw data to mRNA expression of subunit of NADPH oxidase (NOX2), 8-Isoprostane level, superoxide dismutase (SOD) enzyme activity, glutathione level of the rats (Figure 3, main document); n=4-6; outliers were excluded based on GraphPad QuickCalcs.

NOX2 mRNA expression (fold, control)			
	**Control**	**MI**	**MI+R**
**Mean±SEM (pooled sample)**	1.00±0.09	2.22±0.34	0.52±0.21
**8-Isoprostane level (pg/ml/mg protein)**			
	**Control**	**MI**	**MI+R**
**n1**	0.41	0.29	0.39
**n2**	0.43	0.33	0.40
**n3**	0.40	0.32	0.36
**n4**	0.42	0.35	0.39
**n5**	0.43	0.35	0.41
**n6**	0.43	0.34	0.42
**SOD enzyme activity (U/mg protein)**			
	**Control**	**MI**	**MI+R**
**n1**	0.033	0.027	0.027
**n2**	0.035	0.026	0.032
**n3**	0.034	0.026	0.028
**n4**	0.034	0.027	0.031
**n5**	0.035	0.029	0.034
**n6**	0.036	0.030	0.034
**GSH level (mmol/mg protein)**			
	**Control**	**MI**	**MI+R**
**n1**	0.31	0.46	0.39
**n2**	0.16	0.67	0.31
**n3**	0.14	0.57	0.33
**n4**	0.27	0.56	0.42
**n5**	0.15	0.58	
**n6**	0.29	0.46	

EXCLI Journal 2019;18:876-892 – ISSN 1611-2156
Received: September 06, 2019, accepted: September 23, 2019, published: September 26, 2019

Supplementary Table 5: Raw data to cardiomyocyte cross-sectional area, collagen %, mRNA expression of atrial natriuretic peptide (ANP), brain natriuretic peptide (BNP), collagen 1, and collagen 3 of the rats (Figure 4, main document); n=4-6; outliers were excluded based on GraphPad QuickCalcs.

Cardiomyocyte cross-sectional Area (µm²)			
	**Control**	**MI**	**MI+R**
**n1**	12674.96	13655.14	11588.70
**n2**	12356.42	14711.00	11817.88
**n3**	10989.00	17331.58	11359.94
**n4**	11055.54	12894.80	12248.26
**n5**	10760.22	14077.18	12693.14
**n6**	13378.10	17571.72	14246.36
**Collagen (%)**			
	**Control**	**MI**	**MI+R**
**n1**	0.11	12.59	8.29
**n2**	0.13	12.23	6.93
**n3**	0.17	14.87	8.37
**n4**	0.11	16.00	10.30
**n5**	0.13	14.81	10.01
**n6**	0.19	13.43	7.03
**ANP (fold, control)**			
	**Control**	**MI**	**MI+R**
**Mean±SEM (pooled sample)**	1.00±0.54	6.20±4.80	4.41±1.97
**BNP (fold, control)**			
	**Control**	**MI**	**MI+R**
**Mean±SEM (pooled sample)**	1.00±0.53	2.20±0.44	0.56±0.21
**Collagen 1 (fold, control)**			
	**Control**	**MI**	**MI+R**
**Mean±SEM (pooled sample)**	1.00±0.11	4.71±0.94	3.51±1.43
**Collagen 3 (fold, control)**			
	**Control**	**MI**	**MI+R**
**Mean±SEM (pooled sample)**	1.00±0.65	8.73±1.73	1.21±0.64

EXCLI Journal 2019;18:876-892 – ISSN 1611-2156
Received: September 06, 2019, accepted: September 23, 2019, published: September 26, 2019

Supplementary Table 6: Raw data to cell viability of H9c2 cell line against roselle extract at different concentration and cellular area of each experimental group in pre-, simultaneous, and post-treatment experiments.(Figure 5, main document); n=3 independent experiments; outliers were excluded based on GraphPad QuickCalcs.

Cell viability (%)									
**Roselle (mg/ml)**	**15**	**10**	**7.5**	**5**	**2.5**	**1.25**	**0.625**	**0.3125**	**0**
**n1**		11.9		78.6	98.4	98.4	106.8	102.9	106.4
**n2**	14.9	7.3	45.5	98.5	136.3	136.3	143.3	129.2	118.6
**n3**	12.3	5.4	39.2	82.6	118.2	118.2	134.9	124.0	129.8

Cellular area pre-treatment (mm^2)						
	**Control**	**AngII**	**10 µg/mL R**	**AngII + 10 µg/mL R**	**AngII + 5 µg/mL R**	**AngII + 1 µg/mL R**
**n1**	0.003226	0.005221	0.003346	0.003093	0.003006	0.002915
**n2**	0.003294	0.005112	0.004128	0.004464	0.004293	0.003287
**n3**	0.004272	0.005742	0.003363	0.004479	0.003776	0.004243
**Cellular area simultaneous-treatment (mm^2)**						
	**Control**	**AngII**	**10 µg/mL R**	**AngII + 10 µg/mL R**	**AngII + 5 µg/mL R**	**AngII + 1 µg/mL R**
**n1**	0.004763	0.007046	0.004072	0.005259	0.003896	0.003629
**n2**	0.003877	0.005358	0.003201	0.003674	0.004084	0.003936
**n3**	0.004322	0.005165	0.00368	0.003022	0.003878	0.003802
**Cellular area post-treatment (mm^2)**						
	**Control**	**AngII**	**10 µg/mL R**	**AngII + 10 µg/mL R**	**AngII + 5 µg/mL R**	**AngII + 1 µg/mL R**
**n1**	0.003705	0.004282	0.003772	0.003602	0.00375	0.003673
**n2**	0.003106	0.005045	0.003136	0.003324	0.003678	0.003566
**n3**	0.004212	0.005438	0.003023	0.002887	0.00261	0.003084

EXCLI Journal 2019;18:876-892 – ISSN 1611-2156
Received: September 06, 2019, accepted: September 23, 2019, published: September 26, 2019

Supplementary Table 7: Raw data to plasma troponin T of the rats. (Supplementary Figure 1, supplementary document); n=6

Troponin T (pg/ml)		
	**Control**	**MI**
**n1**	209.38	419.22
**n2**	286.09	371.41
**n3**	234.38	410.94
**n4**	223.91	503.13
**n5**	273.28	387.50
**n6**	218.13	346.09

Supplementary Table 8: Raw data to cellular area of H9c2 cell line with 24H incubation of angiotensin II. (Supplementary Figure 2, supplementary document); n=2 independent experiments; outliers were excluded based on GraphPad QuickCalcs.

Cellular area (mm^2)			
	**Control**	**100 nM Ang II**	**200 nM Ang II**
**n1**	0.0059	0.00740	0.00960
**n2**	0.0063	0.00740	0.00970

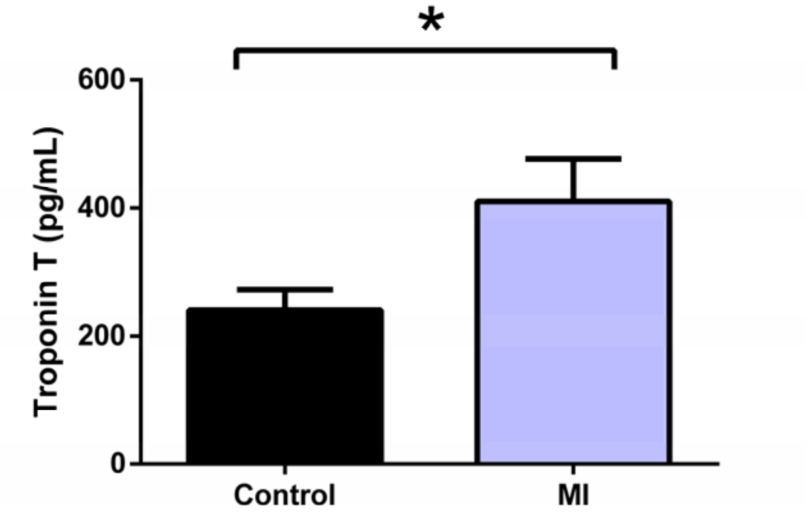

Supplementary Figure 1: Plasma level of troponin T for validation of myocardial infarction. Values are presented as mean ± SEM for n=6 per group. $^{a}p < 0.05$ vs. Control

EXCLI Journal 2019;18:876-892 – ISSN 1611-2156
Received: September 06, 2019, accepted: September 23, 2019, published: September 26, 2019

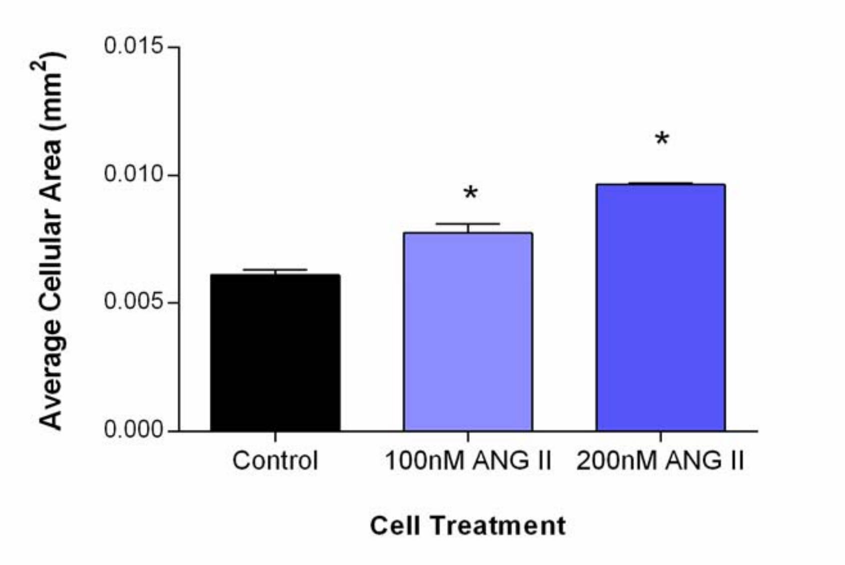

Supplementary Figure 2: Impact of ANG II on cardiomyocyte size after 24 h incubation. Values are presented as mean ± SEM for n=3 independent experiments. [a] $p < 0.05$ vs. Control
